# Characterization and genome analysis of lytic *Vibrio* phage VPK8 with potential in lysing *Vibrio parahaemolyticus* isolates from clinical and seafood sources

**DOI:** 10.1186/s12985-025-02637-6

**Published:** 2025-01-30

**Authors:** Valalak Jintasakul, Jiranan Pattano, Sutima Preeprem, Pimonsri Mittraparp-arthorn

**Affiliations:** 1 Division of Biological Science, Faculty of Science, Prince of Songkla University, Hat Yai, Songkhla 90110 Thailand; 2 Center of Research and Innovation Development of Microbiology for Sustainability (RIMS), Faculty of Science, Prince of Songkla University, Hat Yai, Songkhla 90110 Thailand; 3 Medical and Industrial Microbiology Program, Faculty of Science Technology and Agriculture, Yala Rajabhat University, Yala, 95000 Thailand

**Keywords:** AHPND, Biocontrol agent, Efficiency of plating, Phage therapy, Shrimp aquaculture, *Vibrio parahaemolyticus*

## Abstract

**Background:**

*Vibrio parahaemolyticus* is a marine bacterium causing seafood-associated gastrointestinal illness in humans and acute hepatopancreatic necrosis disease (AHPND) in shrimp. Bacteriophages have emerged as promising biocontrol agents against *V. parahaemolyticus*. This study characterizes *Vibrio* phage VPK8, focusing on host specificity, efficiency of plating (EOP) variability across *V. parahaemolyticus* isolates from diverse sources and other *Vibrio* species, morphology, genomic features, and bacteriolytic potential.

**Methods:**

*Vibrio* phage VPK8 was isolated from blood cockles in Thailand using a mixed-host approach and purified via the double-layer agar method. Host specificity was evaluated using spot assays and EOP measurements against 120 *Vibrio* strains, including AHPND-associated, clinical, and seafood isolates. Phage morphology was characterized by transmission electron microscopy (TEM), while genomic features were analyzed using next-generation sequencing. Lytic characteristics, including latent period and burst size, were determined through one-step growth curves, and bacterial growth reduction was evaluated over a 24-h.

**Results:**

*Vibrio* phage VPK8 is a lytic phage with a 42,866 bp linear double-stranded genome, G + C content of 49.4%, and 48 coding sequences. Phylogenetic analysis grouped it within the *Autographiviridae* family, showing 95.96% similarity to * Vibrio* phage vB_VpaP_MGD1. Viral proteomic analysis placed VPK8 within the *Pseudomonadota* host group. Spot assays indicated broad lytic activity, but EOP analysis revealed high infectivity in clinical and seafood *V. parahaemolyticus* isolates, as well as some *V. cholerae* and *V. mimicus* strains. TEM revealed an icosahedral head (~ 60 nm) and a short tail. At a multiplicity of infection of 0.01, VPK8 exhibited a latent period of 25 min, a burst size of 115, and effectively inhibited the reference host *V. parahaemolyticus* PSU5124 within 6 h, maintaining its lytic activity and stability for over 24 h.

**Conclusions:**

This study provides a detailed characterization of *Vibrio* phage VPK8 which exhibits targeted infectivity with high EOP in clinical and seafood *V. parahaemolyticus* isolates, as well as selected *Vibrio* species. Its stable lytic performance, rapid replication, and genomic safety suggest its potential for phage-based applications. Further studies should explore its in vivo efficacy and the genetic features contributing to phage resistance mechanisms, enhancing its potential applicability in managing *Vibrio*-related diseases.

**Supplementary Information:**

The online version contains supplementary material available at 10.1186/s12985-025-02637-6.

## Background

*Vibrio parahaemolyticus* is a halophilic, Gram-negative bacterium that poses significant challenges to public health and aquaculture. It is a leading cause of seafood-associated gastrointestinal illness in humans and acute hepatopancreatic necrosis disease (AHPND) in shrimp, which has caused severe economic losses in aquaculture since its emergence in 2009 [1]. The major virulence factors of clinical *V. parahaemolyticus* strains are thermostable direct hemolysin (TDH) and TDH-related hemolysin (TRH), which play a critical role in causing foodborne illnesses in humans. In shrimp, AHPND-causing strains (designated VpAHPND) carry an extrachromosomal plasmid, pVA1 (approximately 69 kb), which encodes *Photorhabdus* insect-related (Pir)-like toxins [2–9], responsible for the rapid destruction of shrimp hepatopancreas tissues, leading to high mortality rates of up to 100% [10, 11].

The widespread use of chemicals and antibiotics to control *V. parahaemolyticus* has raised concerns about the development of antibiotic-resistant strains and the accumulation of chemical residues, highlighting the need for alternatives [12]. Bacteriophages, or phages, have shown potential as biocontrol agents due to their host specificity and ability to lyse pathogenic bacteria without disrupting beneficial microbial communities. Recent studies have isolated lytic phages specific to *V. parahaemolyticus*, such as VP41s3 and CAU_VPP01, demonstrating their ability to lyse the bacteria effectively in vitro and in seafood samples [13, 14]. Phages have shown significant efficacy in disrupting biofilms on various surfaces, maintaining seafood quality, and decreasing gene expression related to pathogenicity formed by *V. parahaemolyticus* [14]. Phage PG288 exhibited strong lytic ability, broad host range against *V. parahaemolyticus* and several distinct *Vibrio* species isolated from seafood and environments [15]. However, its infectivity was notably limited against clinical isolates. Furthermore, broad host range *Vibrio* phage OY1 exhibited activity against *V. parahaemolyticus* from different sources and other *Vibrio* species has been investigated [16]. However, its infectivity analysis was limited to isolates from China, excluding shrimp disease-causing strains such as AHPND-associated *V. parahaemolyticus*. In addition to their applications in the food industry, bacteriophages have been employed as biocontrol agents in the aquaculture industry, significantly reducing mass mortality in shrimp and other aquatic species. For example, nucleus-forming *Vibrio* phages isolated from local sources significantly reduced mortality in shrimp infected with VpAHPND isolates [17]. Similarly, *Vibrio* phage vB_VpM-pA2SJ1 was shown to lyse VpAHPND isolates from Latin America, Mexico, and Vietnam, but its limited effectiveness against strains from South Korea and Thailand [18]. Although various studies have demonstrated that lytic bacteriophages serve as effective alternatives for controlling *V. parahaemolyticus*, many have overlooked the inclusion of diverse sources and species. This study isolated and characterized *Vibrio* phage VPK8, a lytic phage that infects *V. parahaemolyticus* and certain strains from 4 *Vibrio* species, including clinical and seafood isolates. The findings from this research could support the development of phage-based strategies for managing *Vibrio*-related diseases in aquaculture and beyond.

## Methods

### Bacteria cultivation

The AHPND, clinical, and seafood-associated *V. parahaemolyticus* strains, along with other *Vibrio* spp. used in this study **(**Table 1**)** were recovered from the − 80 °C culture collection of the Division of Biological Science (Microbiology), Faculty of Science, Prince of Songkhla University, Thailand. The strains were cultured on trypticase soy agar (TSA) supplemented with 1% NaCl and incubated at 30 °C for 16–18 h. Then, 1–2 colonies were inoculated into 3 mL of Trypticase soy broth (TSB) supplemented with 1% NaCl, incubated in a shaking incubator at 150 rpm and 30 °C for 4–5 h, and adjusted to a turbidity of 0.5 McFarland for use in further experiments.

### Sample preparation

*Vibrio* phage VPK8 was isolated from fresh blood cockles (*Tegillarca granosa*) obtained from a local market in Songkhla Province, Thailand, following the method described by Tan et al. [19] with slight modifications. Briefly, 10 g of sample was crushed and transferred into 10 mL of SM buffer in a 50 mL Falcon tube. The mixture was shaken at 150 rpm for 15 min, centrifuged at 10,000 × g for 10 min, and the supernatant was filtered through a 0.2 μm pore size filter. The filtrate was then mixed with 200 µL of a log-phase culture of mixed *V. parahaemolyticus* strains (PSU3866, PSU4413, and PSU5124) and incubated at 30 °C, 150 rpm for 6 h in an incubator shaker. After incubation, the mixture was centrifuged at 10,000 × g for 5 min, and the supernatant was filtered through a 0.2 μm pore size filter.

### *Vibrio* phage isolation and purification

Phage isolation was performed using the double-layer method. Briefly, 200 µL of enriched phage was mixed with 200 µL of a *V. parahaemolyticus* host culture, transferred into 3 mL of soft agar (tryptic soy broth supplemented with 1% NaCl and 0.7% agar), and overlaid onto TSA supplemented with 1% NaCl. After the agar solidified, the plates were incubated at 30 °C for 16 h. Plaques were collected, resuspended in 500 µL of SM buffer, and stored at 4 °C. Phages were purified through three rounds of single-plaque isolation using the double-layer agar method. For this, 3 mL of SM buffer was poured onto the plaque-containing agar surface and incubated at 30 °C, 150 rpm for 16 h. The SM buffer and soft agar were collected, centrifuged at 10,000 × g for 5 min, filtered through a 0.22 μm pore size filter, and stored at 4 °C for subsequent experiments.

### Host range determination

The host range of *Vibrio* phage VPK8 was evaluated using 120 strains of *Vibrio* spp., including AHPND-associated, clinical, and seafood isolates of *V. parahaemolyticus*, as well as other pathogenic species, including *V. alginolyticus*, *V. campbellii*, *V. cholerae*, *V. fluvialis*, *V. harveyi*, *V. mimicus*, and *V. vulnificus*. For each strain, 200 µL of a log-phase bacterial culture (0.5 McFarland) was mixed with 3 mL of soft agar and overlaid onto TSA supplemented with 1% NaCl. After the agar solidified, *Vibrio* phage VPK8 at a concentration of approximately 10^7^-10^8^ PFU/mL was spotted onto the bacterial lawn and incubated at 30 °C for 16 h. Clear zones indicating lysis plaques were observed.

### Determination of efficiency of plating (EOP)

All bacterial strains susceptible to *Vibrio* phage VPK8 in spot assays were further evaluated for productive infection through efficiency of plating (EOP) analysis, following previously described methods with some modifications [20]. Briefly, *Vibrio* phage VPK8 suspension was serially diluted and spotted on test strains as well as the reference host strain, *V. parahaemolyticus* PSU5124. EOP was calculated as the ratio of plaque-forming units (PFU) on test strains to PFU on the reference host strain. Results were categorized as high (EOP > 0.1), moderate (0.005 < EOP  < 0.099), low (EOP < 0.005), or inefficient (no plaques detected) [21].

### Transmission electron microscopy (TEM)

The morphology of *Vibrio* phage VPK8 was examined using a Transmission Electron Microscope (TEM). A 5 µL sample of freshly prepared *Vibrio* phage VPK8 (approximately 10^8^ PFU/mL) was dropped on a carbon-coated grid and stained with 1% uranyl acetate for 2 min. After drying, the grid was analyzed using a Talos™ F200i TEM operated at 200 kV with a magnification of ×94,000.

### Genome sequencing, annotation, and bioinformatic analysis

The genomic DNA of *Vibrio* phage VPK8 was extracted using a phage DNA extraction kit (Norgen Biotek, Thorold, Ontario). The concentration and quality of the extracted DNA was determined using a NanoDrop spectrophotometer (Maestrogen, Inc., Nevada, USA) and confirmed by electrophoresis on a 1% agarose gel with a 1 kb ladder as the marker [22]. Whole-genome sequencing was performed by Macrogen, Inc. (Seoul, South Korea) using an Illumina system. Quality control of the reads was conducted using FastQC, and de novo assembly was carried out using SPAdes. Genome annotation was performed with Rapid Annotation using Subsystem Technology (RAST), and protein analysis was conducted using BLASTP against the NCBI database. VirulenceFinder version 2.0 [23], Resfinder 4.6.0 [24], and PhageLeads [25] were used to identify virulence genes, antibiotic resistance genes, and temperate phage markers, respectively. The lifestyle of *Vibrio* phage VPK8 was determined using PhaBox with default parameter settings [26].

### Phylogenetic tree analysis

The evolutionary relationships of *Vibrio* phage VPK8 with other phages were analyzed using whole-genome sequences and protein alignments. Phylogenetic tree construction was based on sequence similarity searches and alignments. Whole-genome sequence comparisons were performed using MegaBLAST, and protein sequences of RNA polymerase, terminase large subunit, and tail fiber proteins were analyzed using BLASTp. Alignments were conducted using ClustalW [27], followed by phylogenetic tree construction with the neighbor-joining method and 1,000 bootstrap replications in MEGA X [28]. Intergenomic similarities among phages were quantified and visualized in a heatmap generated via VIRIDIC [29], providing insights into genomic relatedness. Genome alignment was performed using the progressiveMAUVE algorithm in Geneious Prime 2024.0 [30]. Comparative genomic relationships were further visualized using the Viral Proteomic Tree (ViPTree) web server under default parameters [31].

### Optimum MOI determination

The optimal multiplicity of infection (MOI) for *Vibrio* phage VPK8 was determined by mixing the phage with a representative isolate, *V. parahaemolyticus* PSU5124, at various MOIs (0.001, 0.01, 0.1, 1, 10, 100, and 1000). The mixtures were incubated at 30 °C, 150 rpm for 3.5 h. Serial dilutions (10^0^ to 10^11^), of the mixtures were prepared, and phage titers were determined using the spot test method. The MOI yielding the highest phage titer was considered optimal.

### One-step growth assay

To study the replication dynamics of *Vibrio* phage VPK8, 10 mL of *V. parahaemolyticus* PSU5124 in the log phase (0.5 McFarland) was mixed with phage at an MOI of 0.01. Samples were collected every 5 min for 2 h, and the phage titer was determined using the spot test method. Plates were incubated at 30 °C for 16 h, and the burst size was calculated.

### Bacterial growth reduction assay

The efficacy of *Vibrio* phage VPK8 in reducing bacterial growth was assessed at various MOIs. Log-phase *V. parahaemolyticus* PSU5124 (0.5 McFarland) was mixed with *Vibrio* phage VPK8 in a 1:1 ratio at MOIs ranging from 0.001 to 1000. Mixtures were transferred to a 96-well plate, and bacterial growth was monitored at 600 nm (OD_600_) every hour for 24 h using a microplate reader.

### Statistical analysis

All data are presented as means ± standard deviations. Statistical analyses were performed using SPSS version 25, with significance set at *P* < 0.05.

## Results

### Isolation and morphological characterization of *Vibrio* phage VPK8

**Fig. 1:**
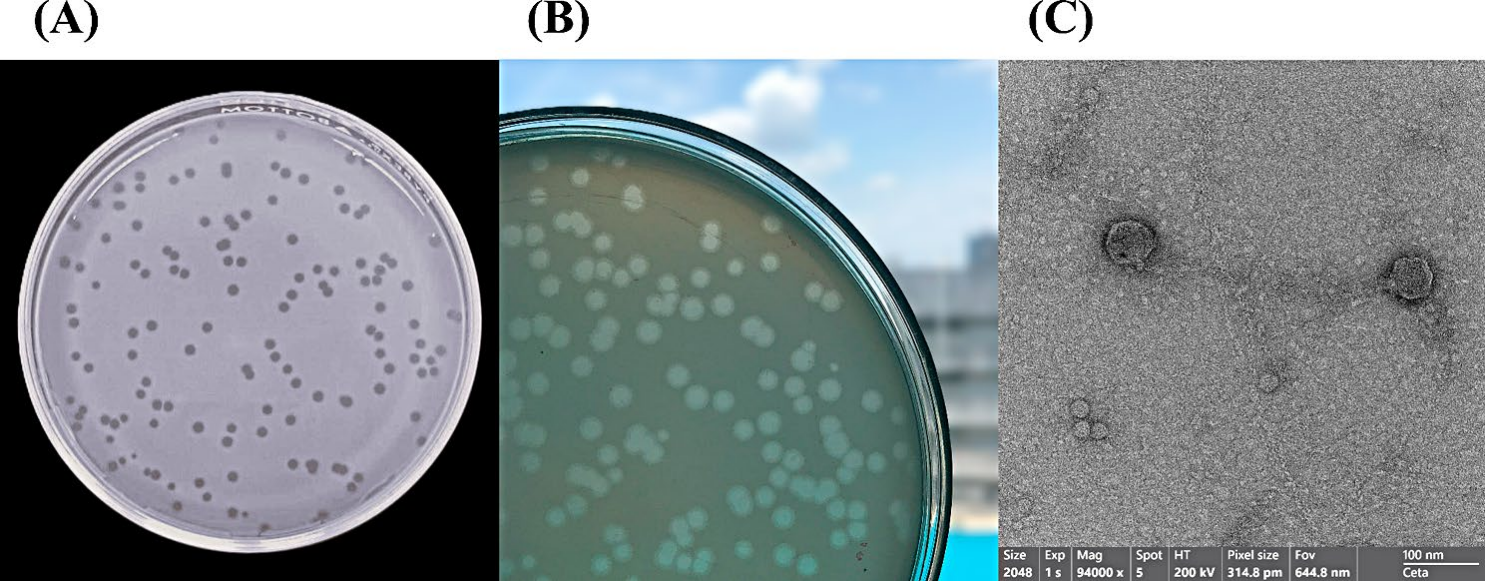
Morphological characterization of *Vibrio* phage VPK8. (**A**) Plaque morphology of *Vibrio* phage VPK8 on *V. parahaemolyticus* PSU5124, forming distinct plaques. (**B**) Halo zones surrounding plaques, indicative of depolymerase activity. (**C**) Transmission electron microscopy (TEM) image of *Vibrio* phage VPK8 showing an icosahedral head (~60 nm in diameter) and a short tail

*Vibrio* phage VPK8 was successfully isolated from blood cockles obtained from a local market in Songkhla Province, Thailand, using triplicate single-plaque isolation. Among the tested strains, *V. parahaemolyticus* PSU5124 produced large, clear plaques, making it the preferred host for subsequent experiments. The plaques formed on a lawn of *V. parahaemolyticus* PSU5124 host bacteria were clear and distinct, indicative of lytic activity (Fig. 1A). These plaques, characterized as “plaques with halos” (Fig. 1B), measured approximately 1.6 mm in diameter. Morphological analysis by TEM revealed that *Vibrio* phage VPK8 possessed a head-tail structure with an icosahedral head measuring approximately 60 nm in diameter and a short, non-contractile tail (Fig. 1C). Based on these features, *Vibrio* phage VPK8 was classified within the family *Autographiviridae*.

### Host range and efficiency of plating (EOP)

The host specificity of *Vibrio* phage VPK8 was evaluated by examining its ability to attach to bacterial receptors and induce lytic activity. The presence of a lysis zone indicated lytic activity, while the absence of a zone indicated no lytic activity or specificity towards that tested bacterial strain. Among the 120 *Vibrio* spp. strains tested, *Vibrio* phage VPK8 exhibited specificity for *V. parahaemolyticus*, inhibiting 58.82% (40/68) of the *V. parahaemolyticus* strains, including 64% (16/25) of VpAHPND strains, 58.33% (14/24) of clinical strains, and 52.63% (10/19) of seafood strains. Furthermore, *Vibrio* phage VPK8 displayed lytic activity against 6 strains of *V. alginolyticus*, 4 strains of *V. campbellii*, 1 strain of *V. cholerae*, and 1 strain of *V. mimicus* (Table 1). However, the efficiency of plating (EOP) analysis revealed that nine strains were highly susceptible to *Vibrio* phage VPK8, with EOP values ranging from 0.273 to 1.255 (Table 2). *Vibrio* phage VPK8 demonstrated effectiveness in forming plaques on most clinical isolates and approximately half of the seafood isolates of *V. parahaemolyticus*, as well as on some isolates of *V. alginolyticus*, *V. cholerae*, and *V. mimicus*. However, all AHPND-associated *V. parahaemolyticus* and *V. campbellii* isolates exhibited inefficient plaque formation, indicating limited infectivity against these strains.

**Table 1:** Host range analysis of *Vibrio* phage VPK8 against 120 strains of *Vibrio* spp.

Bacterial species (*n*)	Isolates	Lytic activity	Reference or source
*V. parahaemolyticus* (68)			
AHPND-associated isolates (25)	PSU5429	+	[32]
	PSU5435, PSU5442, PSU5492, PSU5495, PSU5544, PSU5554, PSU5565, PSU5567, PSU5575, PSU5600, PSU5611, PSU5612, PSU5613, PSU5614, PSU5616	+	Laboratory collection
	PSU5499, PSU5580, PSU5591	-	[32]
	PSU5532, PSU5539, PSU5549, PSU5564, PSU5581, PSU5609	-	Laboratory collection
Clinical isolates (24)	PSU1031, PSU3866, PSU3872, PSU3949, PSU5126	+	[33]
	PSU3380, PSU4915, PSU4943, PSU4994, PSU5190, PSU5194, PSU5257	+	Laboratory collection
	PSU3952	+	[34]
	ATCC17802	+	ATCC
	PSU3328, PSU3352, PSU3536, PSU3906, PSU3918, PSU3937, PSU4286, PSU4921, PSU4956, PSU5055	-	[33]
Seafood isolates (19)	PSU2467, PSU3365, PSU4055, PSU4058, PSU4425, PSU4459, PSU4460, PSU4888, PSU5124, PSU5382	+	[35]
	PSU2463, PSU2471, PSU3819, PSU3831, PSU4062, PSU4413, PSU4415, PSU4869, PSU4879	-	[35]
*V. alginolyticus* (11)	*PSU4537*	+	[36]
	PSU5637, PSU5638, PSU5639, PSU5640, PSU5641	+	Laboratory collection
	PSU4110, PSU4111, PSU4246, *PSU4543*	-	[36]
	*PSU4719*	-	Laboratory collection
*V. campbellii* (13)	PSU6519, PSU6523, PSU6525, PSU6526	+	Laboratory collection
	HY01	-	[37]
	PSU6503, PSU6504, PSU6506, PSU6507, PSU6508, PSU6510, PSU6511, PSU6527	-	Laboratory collection
*V. cholerae* (18)	PSU6161	+	Laboratory collection
	*PSU3662*, *PSU3664*, *PSU3789*, *PSU4711*, PSU5632, PSU5633, PSU5634, PSU5635, PSU5636, PSU6163, PSU6164, PSU6173, PSU6174, PSU6183, PSU6184, PSU6193, PSU6194	-	Laboratory collection
*V. fluvialis* (2)	PSU5701, PSU5704	-	Laboratory collection
*V. mimicus* (1)	*PSU3426*	+	Laboratory collection
*V. vulnificus* (7)	PSU5703, PSU5706	-	Laboratory collection
	C35, D22	-	[38]
	PSU025, PSU039	-	(39)
	DMST 31752	-	DMST

+, *Vibrio* phage VPK8 showed lysis zone; -, *Vibrio* phage VPK8 not showed lysis zone

ATCC, American Type Culture Collection; DMST, Department of Medical Sciences Thailand

For non-*V. parahaemolyticus* isolates: *Underline*, bacteria were isolated from clinical sources; Normal text, bacteria were isolated from animal sources

**Table 2:** Efficiency of plating of *Vibrio* phage VPK8 against 52 phage-sensitive *Vibrio *spp.

Bacterial species (*n*)	Isolates	EOP value	EOP rank	Bacterial species (*n*)	Isolates	EOP value	EOP rank
AHPND-associated *V. parahaemolyticus* (16)	PSU5429 PSU5435 PSU5442 PSU5492 PSU5495 PSU5544 PSU5554 PSU5565 PSU5567 PSU5575 PSU5600 PSU5611 PSU5612 PSU5613 PSU5614 PSU5616	0 0 0 0 0 0 0 0 0 0 0 0 0 0 0 0	Inefficient Inefficient Inefficient Inefficient Inefficient Inefficient Inefficient Inefficient Inefficient Inefficient Inefficient Inefficient Inefficient Inefficient Inefficient Inefficient	Seafood isolates of *V. parahaemolyticus* (10)	PSU2467 PSU3365 PSU4055 PSU4058 PSU4425 PSU4459 PSU4460 PSU4888 PSU5124 PSU5382	0 0 0.024 0 0.527 0.024 0.062 0 1.000 0	Inefficient Inefficient Moderate Inefficient High Moderate Moderate Inefficient High Inefficient
Clinical isolates of *V. parahaemolyticus* (14)	PSU1031 PSU3380 PSU3866 PSU3872 PSU3949 PSU3952 PSU4915 PSU4943 PSU4994 PSU5126 PSU5190 PSU5194 PSU5257 ATCC17802	0 0 0.296 0.002 0.062 0 0.273 0.073 1.255 0.002 0 0.387 0.031 0	Inefficient Inefficient High Low Moderate Inefficient High Moderate High Low Inefficient High Moderate Inefficient	*V. alginolyticus* (6)	*PSU4537* PSU5637 PSU5638 PSU5639 PSU5640 PSU5641	0 0.745 0 0 0 0.001	Inefficient High Inefficient Inefficient Inefficient Low
				*V. campbellii* (4)	PSU6519 PSU6523 PSU6525 PSU6526	0 0 0 0	Inefficient Inefficient Inefficient Inefficient
				*V. cholerae* (1)	PSU6161	1.109	High
				*V. mimicus* (1)	*PSU3426*	0.327	High

ATCC, American Type Culture Collection; DMST, Department of Medical Sciences Thailand

For non-*V. parahaemolyticus* isolates: *Underline*, bacteria were isolated from clinical sources; Normal text, bacteria were isolated from animal sources

### Genomic features of *Vibrio* phage VPK8

**Fig. 2:**
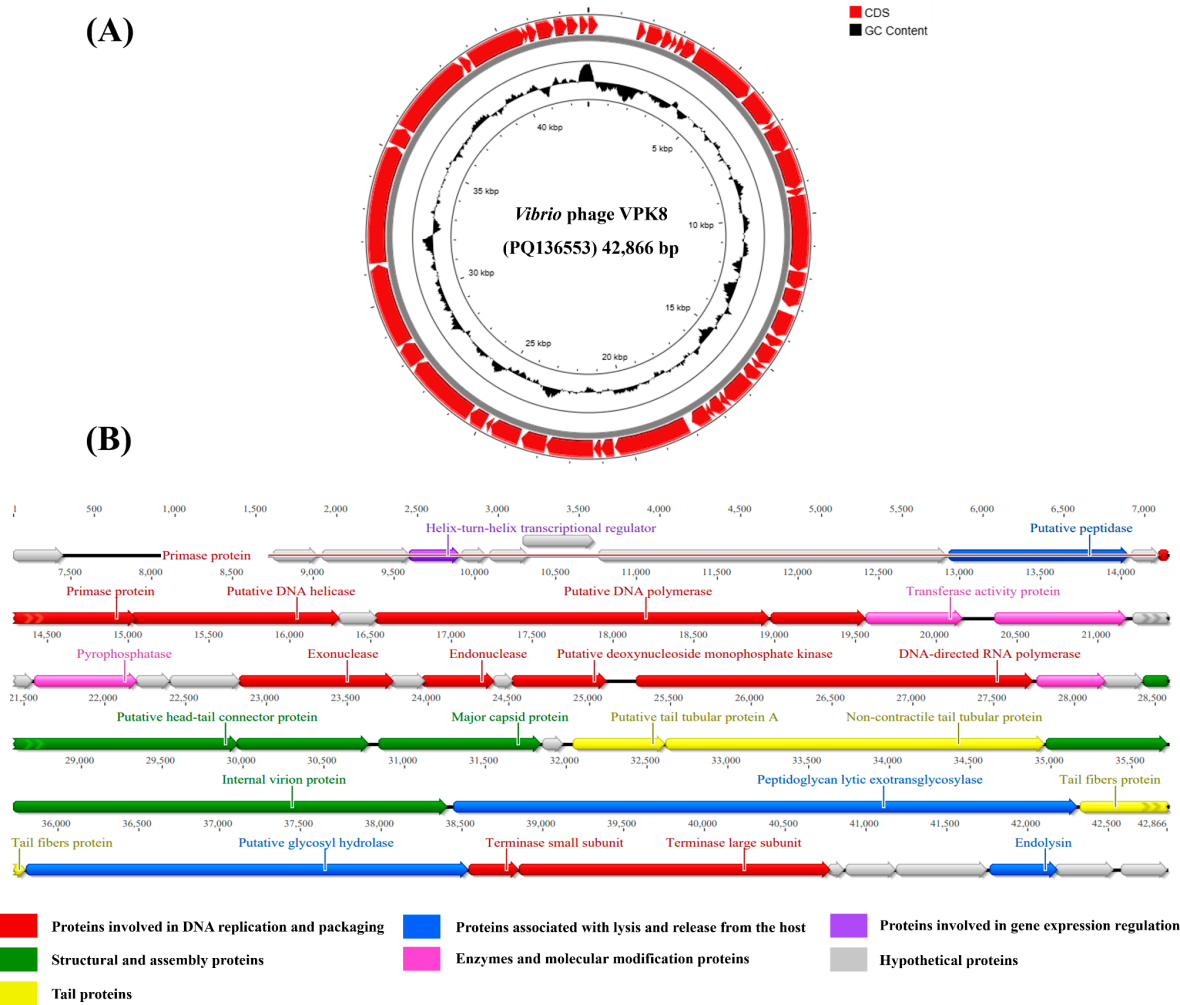
Genomic organization of *Vibrio* phage VPK8. (**A**) Circular genome map showing the GC content (innermost circle, black) and coding sequences (outer circle, red). (**B**) Genomic features categorized by function

The whole genome of *Vibrio* phage VPK8 was analyzed to investigate its structure, gene content, and protein functions. The analysis revealed a linear double-stranded DNA genome of 42,866 bp with a GC content of 49.4% (Fig. 2A). The genome encodes 48 coding sequences (CDSs), of which 27 have been functionally annotated, while the remaining 21 CDSs are hypothetical proteins with unknown functions (Additional file 1: Table S1). Functionally categorized proteins were classified into 7 categories: proteins involved in DNA replication and packaging, structural and assembly proteins, tail proteins, proteins associated with lysis and release from the host, enzymes and molecular modification proteins, proteins involved in gene expression regulation, and hypothetical proteins, all located on the positive strand (Fig. 2B).

Notably, the absence of tRNAs, virulence factor genes, toxin genes, antibiotic resistance genes, and integrase genes suggests that *Vibrio* phage VPK8 adopts a lytic life cycle. This classification was further confirmed using PhaBox, which identified VPK8 as a virulent lifestyle phage.

### Phylogenetic and comparative genomic analyses

**Fig. 3:**
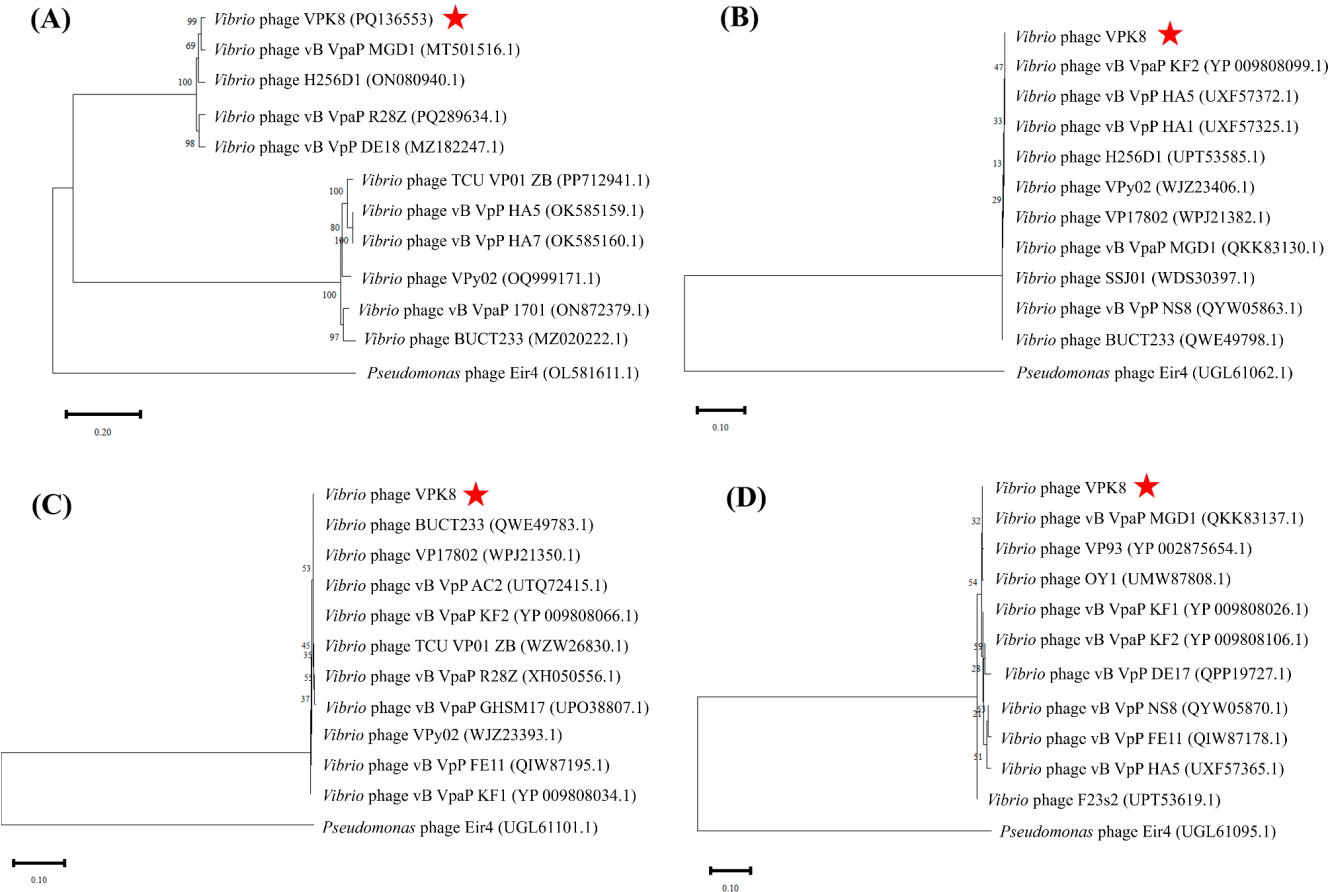
Phylogenetic analysis of *Vibrio* phage VPK8. Neighbor-joining phylogenetic trees based on (**A**) whole genome sequences, (**B**) RNA polymerase, (**C**) terminase large subunit, and (**D**) tail fiber protein sequences. The analysis reveals close evolutionary relationships between *Vibrio* phage VPK8 and related phages. Trees were constructed with 1,000 bootstrap replicates using MEGA X

Phylogenetic tree construction based on whole-genome sequence similarity positioned *Vibrio* phage VPK8 as closely related to *Vibrio* phage vB_VpaP_MGD1, a member of the *Autographiviridae* family, with a genomic identity of 95.96% (Fig. 3A). Protein-based phylogenies constructed from RNA polymerase, terminase large subunit, and tail fiber protein alignments further confirmed the phylogenetic placement of *Vibrio* phage VPK8. These analyses demonstrated high bootstrap support, grouping it with lytic *Vibrio* phages. Notably, the RNA polymerase phylogeny revealed a close relationship between *Vibrio* phage VPK8 and other members of the *Autographiviridae* family (Fig. 3B). The terminase large subunit phylogeny demonstrated closer relationships to *Vibrio* phage BUCT233 (Fig. 3C), while the tail fiber protein phylogeny indicated a stronger association with *Vibrio* phage vB_VpaP_MGD1 (Fig. 3D), highlighting functional and evolutionary divergence among these related phages.

**Fig. 4:**
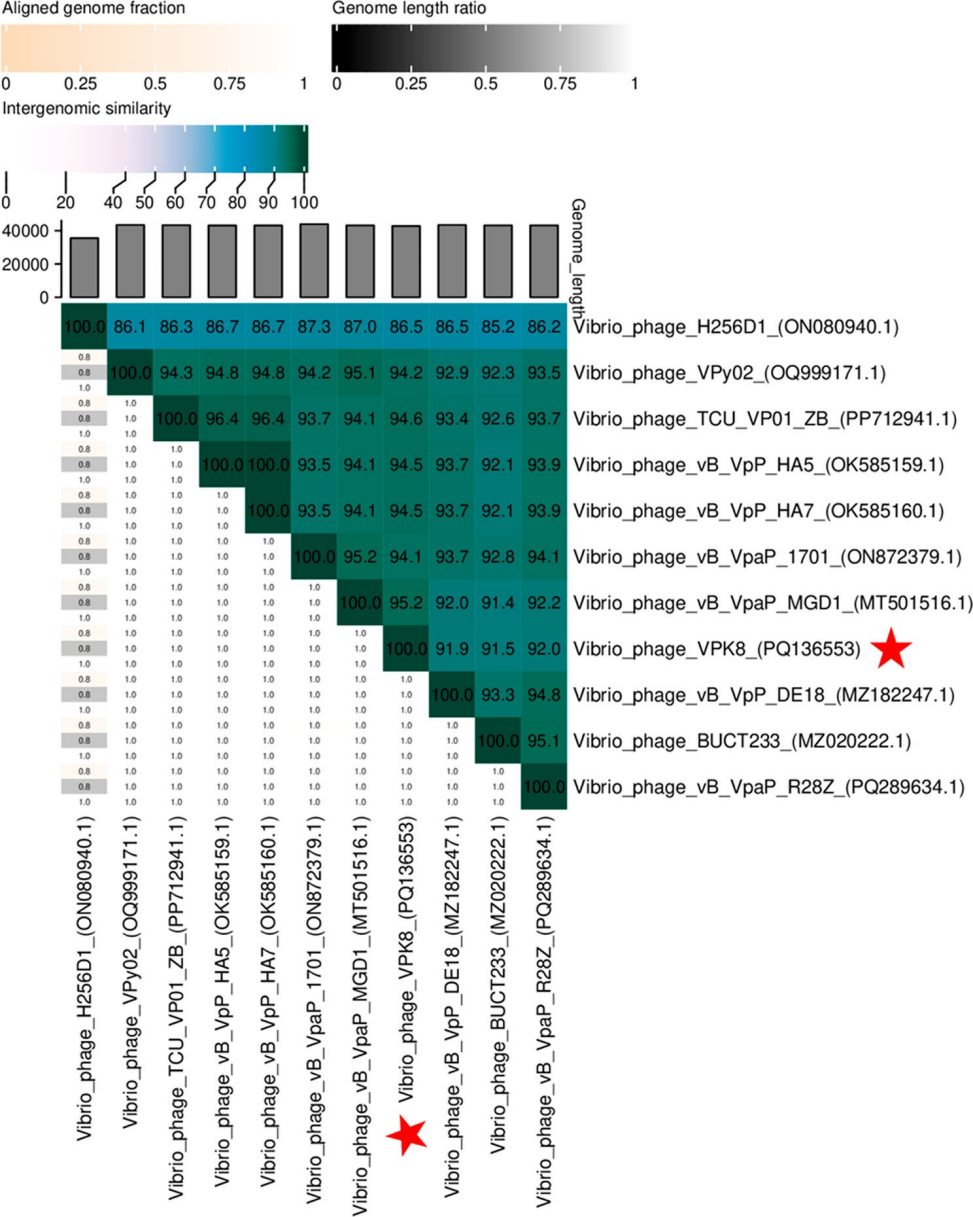
Heatmap showing genomic similarity between *Vibrio* phage VPK8 and other *Vibrio* phages. The color gradient indicates intergenomic similarity: darker colors (green to white) represent higher similarity, while genome length ratio (black to white) and aligned genome fractions (orange to white) highlight structural differences. Analysis was performed using the VIRIDIC tool

**Fig. 5:**
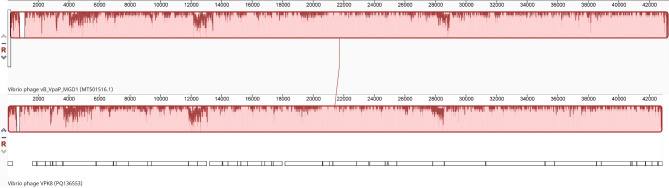
Comparative genome analysis between *Vibrio* phage VPK8 and *Vibrio* phage vB_VpaP_MGD1. Sequence alignment show conserved regions (colored blocks) and unique sequences (non-aligned regions). Analysis was conducted using the progressiveMauve algorithm

Intergenomic similarities results displayed as a heatmap showed that *Vibrio* VPK8 exhibited the highest genomic similarity (95.2%) with *Vibrio* phage vB_VpaP_MGD1 (Fig. 4), while more distant relationships were observed with phages infecting other *Vibrio* species, underscoring its niche specialization. Therefore, these findings place *Vibrio* phage VPK8 in the family *Autographiviridae*, which belongs to Class *Caudoviricetes*. The genomic alignment of VPK8 and vB_VpaP_MGD1 revealed a minor missing gene cluster in VPK8, consistent with the heatmap analysis (Fig. 5).

### Viral proteomic tree analysis

**Fig. 6:**
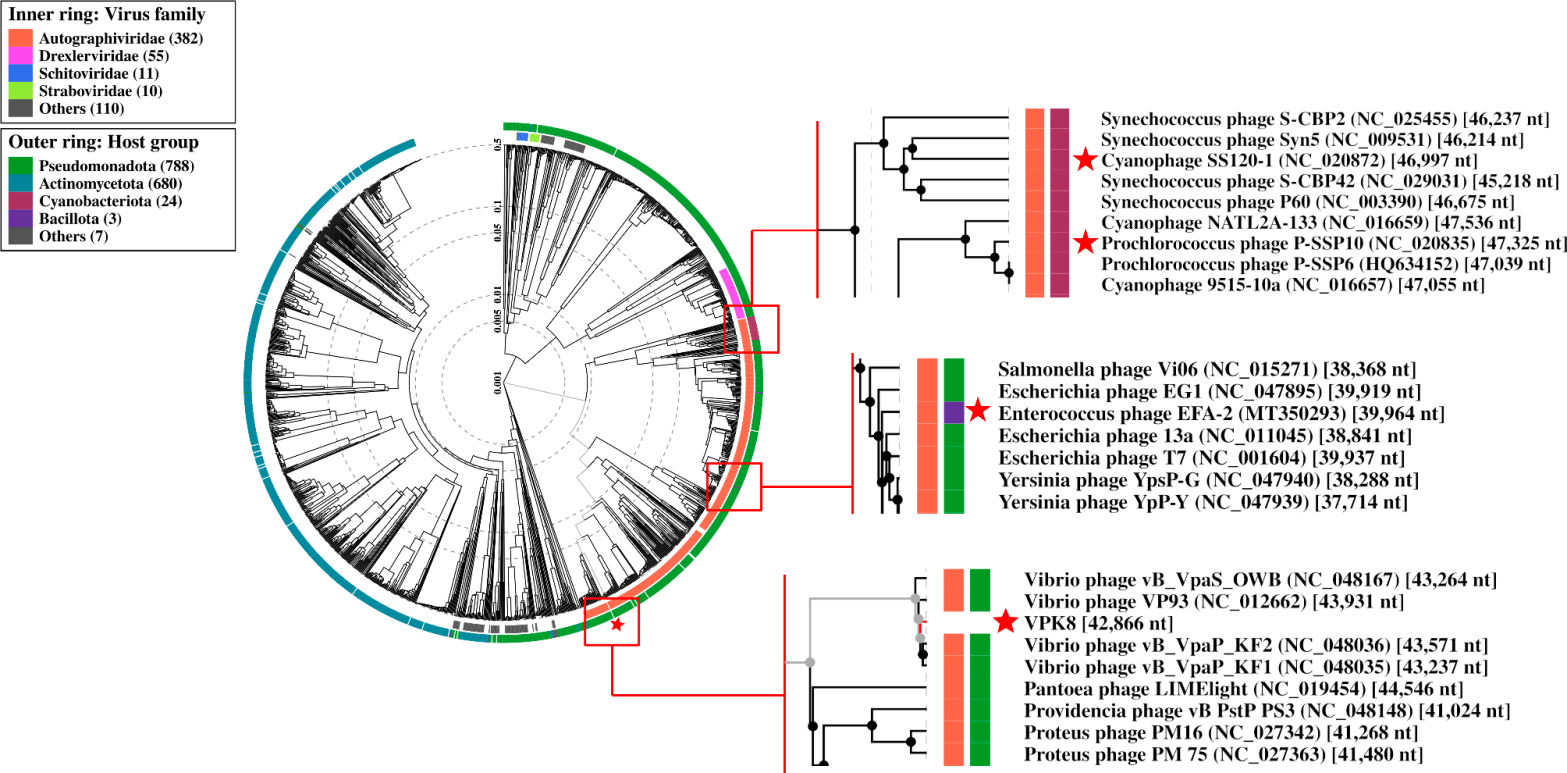
Circular proteomic tree of *Vibrio* phage VPK8. The inner ring indicates the virus families, (e.g., *Autographviridae*), while the outer ring represents host groups, including *Pseudomonata*, *Cyanobacteriota*, and *Bacillota*. *Vibrio* phage VPK8 and comparative phages (e.g. Cyanophage SS120-1, *Prochlorococcus* phage, and *Enterococcus* phage EFA-2 are higlights). are marked with a red star on the rectangular proteomic tree. Tree generated using the ViPTree web server

**Fig. 7:**
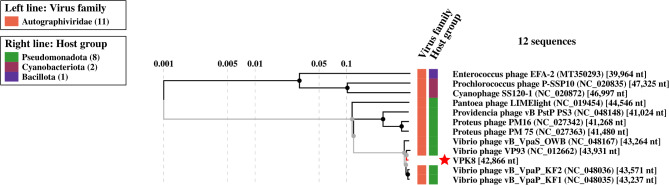
Rectangular proteomic tree *Vibrio* phage VPK8 and related bacteriophages. The tree shows the relationship between virus families (left) and host groups (right). *Vibrio* phage VPK8 is marked with a red star within the *Pseudomonadota* group. Generated using the Vibtree web server

The Viral Proteomic Tree (ViPTree) analysis demonstrated the clustering of VPK8 with phages infecting *V. parahaemolyticus*, reflecting shared evolutionary origins and genomic features (Fig. 6). To further explore the evolutionary placement and host-specific adaptations of *Vibrio* phage VPK8, three phages from the *Autographiviridae* family-*Enterococcus* phage EFA-2, *Prochlorococcus* phage P-SSP10, and Cyanophage SS120-1 representing different host groups were selected for comparison. The ViPTree analysis revealed distinct clustering patterns based on host specialization. *Vibrio* phage VPK8 formed a branch closely related to *Vibrio*-specific phages within the Pseudomonadota (synonym Proteobacteria) host group, emphasizing its ecological niche and evolutionary divergence from phages infecting hosts within Cyanobacteriota and Bacillota (Fig. 7).

### Optimum MOI and host bacterial growth reduction

**Fig. 8:**
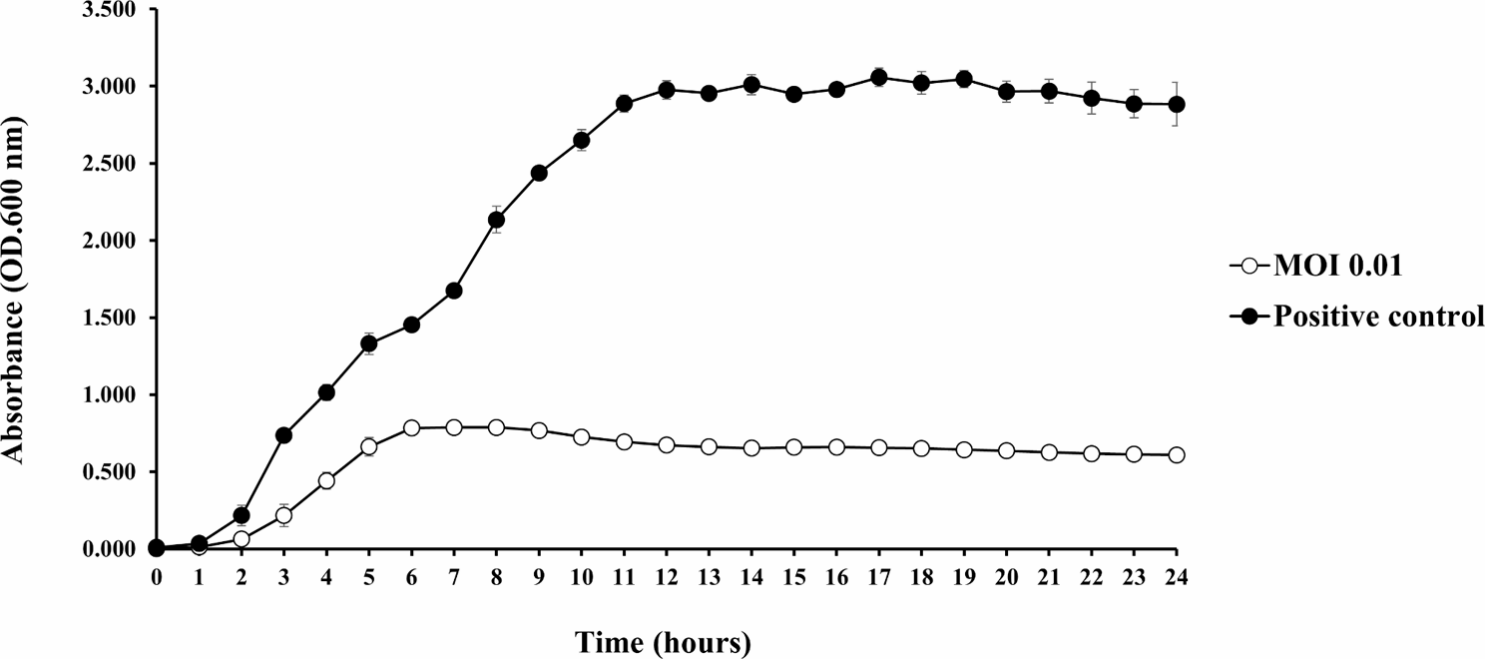
Growth reduction of *V. parahaemolyticus* PSU5124 treated with *Vibrio* phage VPK8. The bacterial growth curve, measured at OD600 over 24 h, shows significant growth suppression in cultures treated with phage at MOI 0.01 compared to controls

The optimal MOI for *Vibrio* phage VPK8 was determined by incubating the phage with log-phase *V. parahaemolyticus* PSU5124 for 3.5 h at MOIs ranging from 0.001 to 1000. An MOI of 0.01 yielded the highest titer of 4.7 × 10^10^ ± 0.06 PFU/mL (Fig. 8), establishing it as the most suitable for further experiments.

**Fig. 9:**
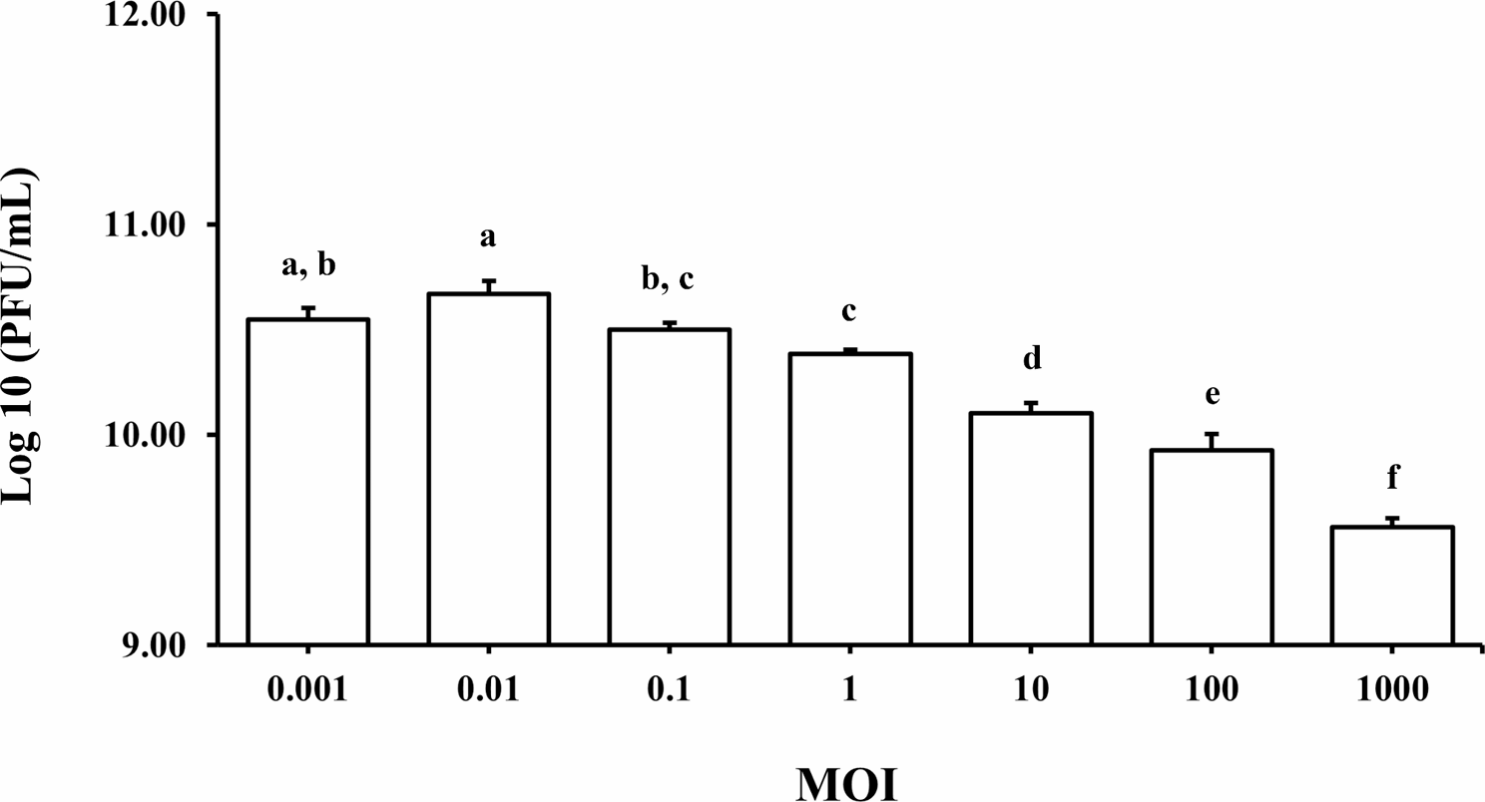
Optimal multiplicity of infection (MOI) of *Vibrio* phage VPK8. Phage efficacy was tested at MOIs ranging from 0.001 to 1000 after 3.5 h of incubation. The experiment identified MOI 0.01 as the most effective for bacteria reduction

The effect of *Vibrio* phage VPK8 on host bacterial growth was performed at an MOI of 0.01 over a 24 h period. The OD of cultures containing phage was significantly lower compared to the control during the first 6 h, indicating effective inhibition of bacterial growth. After this initial period, the OD of phage-treated cultures remained stable, while the control culture without phage exhibited continuous growth, as evidenced by an increase in OD (Fig. 9). These findings highlight the ability of *Vibrio* phage VPK8 to effectively suppress bacterial growth.

### One-step growth curve

**Fig. 10:**
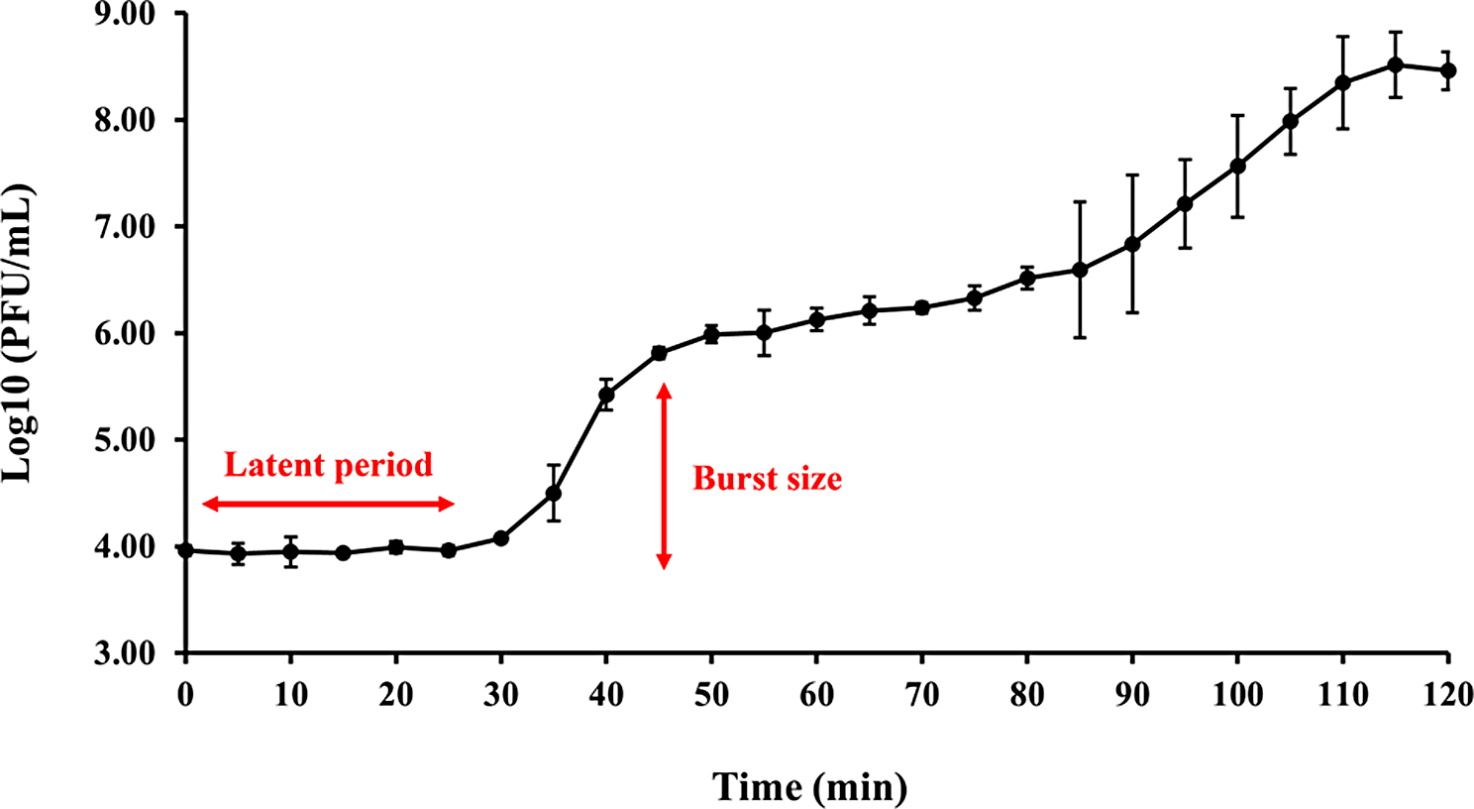
One-step growth curve of *Vibrio* phage VPK8. The replication dynamics of *Vibrio* phage VPK8 were analyzed over a 2-hour period, revealing a 25-min latent phase, a burst size of 115, and high replication efficiency

The replication pattern of *Vibrio* phage VPK8 was investigated using a one-step growth curve experiment at an MOI of 0.01. Following a 10-min adsorption phase, *Vibrio* phage VPK8 entered a latent period lasting approximately 25 min. After this phase, the phage rapidly replicated and began to release from the host bacterial cells, reaching a peak titer at approximately 115 min. The calculated burst size was 115, indicating a high replication capacity within a short period following infection (Fig. 10).

## Discussion

This study characterizes *Vibrio* phage VPK8, a lytic phage isolated from blood cockles in Thailand, highlighting its potential as a biocontrol agent against *V. parahaemolyticus* and related species. VPK8 demonstrated robust lytic activity across a range of *V. parahaemolyticus* isolates, including clinical and seafood strains, but showed limited infectivity toward AHPND-associated strains. These findings emphasize its specificity and underscore the complexity of host-phage interactions. The host range and efficiency of plating (EOP) analyses revealed that VPK8 effectively lysed multiple *Vibrio* species, including some strains of *V. alginolyticus*, *V. cholerae*, and *V. mimicus*. The differential EOP values, ranging from high infectivity in clinical and seafood isolates to inefficiency in AHPND strains, suggest variability in receptor compatibility or resistance mechanisms among *V. parahaemolyticus* strains obtained from various sources and other *Vibrio* species. In addition, although most studies on VpAHPND-phages focus on their application in phage therapy, reporting effects on the survival of *Penaeus vannamei* challenged with *V. parahaemolyticus* causing AHPND, the survival rates often show only modest improvement [17, 40]. This limited efficacy could be attributed to phages inhibiting bacterial growth without fully eradicating the pathogens, possibly due to incomplete lysis or resistance mechanisms. This variability highlights the need to incorporate EOP assays alongside spot tests for accurate phage evaluation, as spot assays alone may overestimate lytic potential [41]. EOP assays are particularly valuable, providing deeper insights into phage-host dynamics and the efficiency of productive infections [20]. in the bacteria.

The ability to lyse certain *V. alginolyticus*, *V. cholerae*, and *V. mimicus* strains underscores the phage’s versatility across different *Vibrio* species. The host range of a bacteriophage is primarily determined by its tail fiber proteins, which enable the phage to identify and attach to specific receptor sites on the surface of the host bacterium [42]. Tail fiber proteins, critical for host recognition, likely play a significant role in VPK8’s host specificity, aligning with findings from similar phages like OY1, which has a broad host range against multiple *Vibrio* species [42]. Further study on the molecular interactions between phage tail proteins and bacterial receptors can inform strategies to engineer phages with tailored host ranges, enhancing their therapeutic potential [43, 44]. Nonetheless, developing a phage cocktail could further expand its host range, enhance its effectiveness against diverse strains, and mitigate the risk of phage resistance while maintaining specificity [45].

The plaques formed by VPK8 displayed halo zones, indicative of depolymerase activity that targets bacterial polysaccharide polymers [46, 47]. Similar halo-forming activity has been observed in phage vABWU2101against multidrug-resistant *Acinetobacter baumannii*, further supporting the hypothesis that depolymerase enzymes enhance bacterial lysis [48]. Genomic analysis of *Vibrio* phage VPK8 revealed the presence of depolymerase-associated genes, such as peptidoglycan lytic exotransglycosylase (PLE), glycosyl hydrolase, and endolysin. These enzymes are crucial for the phage’s lytic activity, as they enable the phage to break down key components of the bacterial cell surface, facilitating bacterial cell wall degradation and promoting effective host lysis. The PLE identified in the phage genome plays a significant role in cleaving the glycosidic bonds between sugar molecules in the peptidoglycan layer of the bacterial cell wall. This enzyme acts by breaking down the structural polysaccharides, thus weakening the bacterial cell wall and allowing for easier penetration and subsequent lysis by the phage [49, 50]. Additionally, glycosyl hydrolases, another class of enzymes found in the VPK8 genome, further contribute to the breakdown of the bacterial surface polysaccharides. These enzymes cleave glycosidic bonds in carbohydrates, which are essential components of the bacterial capsule and other surface polysaccharides that protect the bacteria from environmental stress and immune responses. The presence of these enzymes in VPK8 suggests that the phage could efficiently degrade extracellular polysaccharides, aiding in bacterial cell wall degradation and enhancing phage infectivity [49, 50]. The detection of endolysins in the genome of VPK8 highlights another critical feature that contributes to its lytic nature. Endolysins are enzymes that act directly on the peptidoglycan layer at the end of the phage replication cycle. They are typically synthesized during the late stage of the phage life cycle and are responsible for the final lysis of the bacterial cell by cleaving the peptidoglycan cross-links. This ensures the release of newly formed phage progeny from the host cell, promoting the spread of the infection to neighboring bacteria [49, 50]. The presence of endolysin genes suggests that VPK8 is equipped for rapid bacterial cell destruction, characteristic of phages that follow a lytic lifecycle.

The analysis of genomic features of *Vibrio* phage VPK8 revealed a well-organized genome encoding essential machinery for its lifecycle, including DNA replication and packaging, structural assembly, host specificity, lysis, and regulatory functions. The DNA replication and packaging machinery includes genes encoding DNA helicase, DNA polymerase, and terminase subunits, which are crucial for genome replication and encapsidation. Terminase enzymes package viral DNA into capsids, driven by ATP hydrolysis. They coordinate genome excision, maturation, and translocation, critical for viral assembly [51]. Structural components of *Vibrio* phage VPK8 were represented by genes encoding the major capsid protein, scaffolding protein, and head-tail connector protein, reflecting the robust assembly of the phage particle. In some phages, scaffolding proteins stabilize the capsid’s interior, ensuring proper assembly and function [52]. In addition, the DNA packaging process is initiated by a portal complex that interacts with the capsid and tail, ensuring efficient genome delivery into the host. This mechanism is conserved across various tailed phages, highlighting the evolutionary significance of these structures [53]. Additional genes for enzymes and molecular modifications were also identified in *Vibrio* phage VPK8, such as transferases and Fe-S oxidoreductases. These genes are categorized as Auxiliary Metabolic Genes (AMGs), which facilitate the phage’s ability to manipulate the host environment [54]. AMGs can enhance viral replication and survival, reflecting the phage’s adaptive strategy to optimize the resources of its bacterial host, especially in aquatic environments [55–57]. Regulatory proteins, such as helix-turn-helix (HTH) transcriptional regulators, were identified, providing insights into the control of gene expression during infection.

Morphologically, *Vibrio* phage VPK8 displays characteristics consistent with other members of the *Autographiviridae*, including an icosahedral head and a short tail [58]. The genomic analysis of *Vibrio* phage VPK8 places it within the family *Autographiviridae*, showing high sequence similarity with other phages, such as *Vibrio* phage vB_VpaP_MGD1 (95.96% identity, 99% coverage) and *Vibrio* phage H256D1 (95.34% identity, 82% coverage). Phylogenetic analysis based on the terminase large subunit and tail fiber protein sequences further confirmed the close relationships of VPK8 with *Vibrio* phage vB_VpaP_MGD1 and/or other phages within the *Autographiviridae* family. These phages were isolated from diverse sources across various geographic regions. The shared genetic features and close phylogenetic relationships highlight the broad distribution and evolutionary conservation of *Vibrio* phages in this family. A distinguishing feature of this family is the presence of RNA polymerase, which facilitates rapid replication and efficient bacterial lysis [59]. This suggests that *Vibrio* phage VPK8 may be highly effective at infecting and lysing *Vibrio* species, which is advantageous for potential applications in phage therapy.

The absence of integrase genes, virulence factor genes, toxin genes, and antibiotic resistance genes in VPK8 further supports its classification as a lytic phage [60]. Moreover, the lack of tRNA genes also reinforces the lytic nature of VPK8, as these genes are typically associated with temperate phages that integrate into the host genome [61]. Overall, this indicates that VPK8 does not contribute to horizontal gene transfer, making it a safer option for phage therapy compared to temperate phages [62].

In experiments to determine the optimal MOI, *Vibrio* phage VPK8 demonstrated a burst size of approximately 115 progeny per bacterial cell and a short latent period, indicating its high efficiency in bacterial lysis [63, 64]. Furthermore, VPK8 effectively reduced *V. parahaemolyticus* growth within the first 6 h and maintained suppression for up to 24 h without signs of bacterial resistance. This consistent lytic activity highlights its potential as a reliable biocontrol agent for *V. parahaemolyticus*.

## Conclusion

This study characterizes *Vibrio* phage VPK8, a lytic phage isolated from blood cockles, highlighting its potential as a biocontrol agent against *V. parahaemolyticus*. VPK8 exhibited robust lytic activity against clinical and seafood isolates but showed limited infectivity toward AHPND-associated strains. Genomic analysis classified VPK8 within the family *Autographiviridae*, with essential genes for replication, assembly, lysis, and auxiliary metabolic functions, supporting its ecological adaptability and lytic nature. The presence of depolymerases and endolysins enhances its bacterial cell wall degradation capabilities. Phylogenetic and proteomic analyses clustered VPK8 with *Vibrio*-specific phages, underscoring its ecological niche. With a high burst size, a short latent period, and sustained suppression of *V. parahaemolyticus* growth, VPK8 shows promise as a biocontrol agent. Further studies should explore its in vivo efficacy and the molecular basis of its specificity and resistance mechanisms in AHPND strains.

## Electronic supplementary material

Below is the link to the electronic supplementary material.

Supplementary Material 1: Table S1. Annotation of *Vibrio* phage VPK8 genome. The open reading frames (ORFs) and representative sequences were analyzed through homology searches against protein entries in the GenBank database using BLASTP.

## Data Availability

The sequence data for *Vibrio* phage VPK8 are available in the NCBI database under the accession number PQ136553.

## Abbreviations

AHPND: Acute Hepatopancreatic Necrosis Disease
ATCC: American Type Culture Collection
Bp: Base pairs
CDSs: Coding Sequences
DNA: Deoxyribonucleic Acid
EMS: Early Mortality Syndrome
GC: Guanine-Cytosine content
h: Hours
kV: Kilovolts
mL: Milliliters
min: Minutes
mm: Millimeters
MDR: Multi-drug Resistance
MOI: Multiplicity of Infection
NaCl: Sodium Chloride
NCBI: National Center for Biotechnology Information
nm: Nanometers
OD: Optical Density
PFU: Plaque-forming units
PirA: Photorhabdus insect-related toxin A
PirB: Photorhabdus insect-related toxin B
PLE: Peptidoglycan Lytic Exotransglycosylase
pVA: Plasmid Vibrio-AHPND
RAST: Rapid Annotation using Subsystem Technology
RNA: Ribonucleic Acid
rpm: Revolutions per minute
Spp.: Species
SM buffer: Salt-Magnesium buffer
SPAdes: St. Petersburg genome assembler
SPSS: Statistical Package for the Social Sciences
TDH: Thermostable Direct Hemolysin
TEM: Transmission Electron Microscopy
T3SS1: Type III Secretion System 1
T3SS2: Type III Secretion System 2
TRH: TDH-related Hemolysin
TSA: Tryptic Soy Agar
TSB: Tryptic Soy Broth
tRNAs: Transfer RNAs
µL: Microliters
µm: Micrometers
*V. parahaemolyticus*: *Vibrio parahaemolyticus*
VpAHPND: *Vibrio parahaemolyticus* Acute Hepatopancreatic Necrosis Disease
