# Supplementary material for: Characterization and genome analysis of lytic Vibrio phage VPK8 with potential in lysing Vibrio parahaemolyticus isolates from clinical and seafood sources
Source: Virol J. 2025 Jan 30;22:21. doi: 10.1186/s12985-025-02637-6 (PMC11783711; doi:10.1186/s12985-025-02637-6)
Supplement: Supplementary file 1 — Supplementary Material 1: Table S1. Annotation of Vibrio phage VPK8 genome. The open reading frames (ORFs) and representative sequences were analyzed through homology searches against protein entries in the GenBank database using BLASTP. [file 12985_2025_2637_MOESM1_ESM.pdf]

Table S1 Annotation of *Vibrio* phage VPK8 genome. The open reading frames (ORFs) and representative sequences were analyzed through homology searches against protein entries in the GenBank database using BLASTP.

ORF	Genomic coordinates	Length (bp)	Amino acid	Representative seq [Organism]	Coverage (%)	*E*-value	Identity (%)	Accession no.
1	3-305	303	100	Hypothetical protein [*Vibrio* phage vB_VpaP_C2]	55	1.00E-09	61.82	USL89902.1
2	1612-1875	264	87	Hypothetical protein [*Vibrio* phage vB_VpaP_KF2]	100	1.00E-55	98.85	YP_009808072.1
3	1914-2450	537	178	Hypothetical protein [*Vibrio* phage VP93]	100	1.00E-127	100	YP_002875626.1
4	2453-2764	312	103	Helix-turn-helix transcriptional regulator [*Vibrio* phage vB_VpaS_OWB]	100	1.00E-68	99.03	YP_009948711.1
5	2774-2923	150	49	Hypothetical protein [*Vibrio* phage F23s2]	100	6.00E-23	95.92	UPT53650.1
6	2949-3182	234	77	Hypothetical protein [*Vibrio* phage vB_VpaP_KF1]	100	3.00E-49	100	YP_009808044.1
7	3154-3585	432	143	Hypothetical protein [*Vibrio* phage VP93]	100	2.00E-101	99.3	YP_002875629.1
8	3626-5767	2142	713	Hypothetical protein [*Vibrio* phage vB_Vc_SrVc2]	100	0	97.48	QQM14897.1
9	5793-6896	1104	367	Putative peptidase [*Vibrio* phage VPy02]	100	0	99.18	WJZ23423.1

Table S1 Annotation of *Vibrio* phage VPK8 genome. The open reading frames (ORFs) and representative sequences were analyzed through homology searches against protein entries in the GenBank database using BLASTP (Continued).

ORF	Genomic coordinates	Length (bp)	Amino acid	Representative seq [Organism]	Coverage (%)	*E*-value	Identity (%)	Accession no.
10	6922-7086	165	54	Hypothetical protein [*Vibrio* phage VP93]	100	5.00E-31	100	YP_002875633.1
11	7086-7898	813	270	Primase protein [*Vibrio* phage vB_VpaP_AL-1]	100	0	99.26	UFK26887.1
12	7880-9160	1281	426	Putative DNA helicase [*Vibrio* phage vB_VpaP_KF1]	100	0	100	YP_009808050.1
13	9160-9393	234	77	Hypothetical protein [*Vibrio* phage vB_VpP_3]	100	3.00E-49	97.4	XBO82116.1
14	9386-11821	2436	811	Putative DNA polymerase [*Vibrio* phage vB_VpaP_MGD1]	100	0	99.75	QKK83116.1
15	11835-12413	579	192	Putative nucleotide modification associated domain-containing protein [*Vibrio* phage vB_VpP_HA1]	100	1.00E-138	98.96	UXF57337.1
16	12422-13015	594	197	Transferase activity protein [*Vibrio* phage vB_VpP_AC2]	100	1.00E-140	97.97	UTQ72382.1
17	13216-14031	816	271	Fe-S oxidoreductase [*Vibrio* phage vB_VpP_DE18]	100	0	100	QWY13555.1

Table S1 Annotation of *Vibrio* phage VPK8 genome. The open reading frames (ORFs) and representative sequences were analyzed through homology searches against protein entries in the GenBank database using BLASTP (Continued).

ORF	Genomic coordinates	Length (bp)	Amino acid	Representative seq [Organism]	Coverage (%)	*E*-value	Identity (%)	Accession no.
18	14077-14409	333	110	Hypothetical protein [*Vibrio* phage vB_VpP_NS8]	100	9.00E-76	100	QYW05854.1
19	14421-15050	630	209	Pyrophosphatase [*Vibrio* phage VPy02]	100	3.00E-150	100	WJZ23415.1
20	15050-15253	204	67	Hypothetical protein [*Vibrio* phage vB_VpaP_MGD1]	100	1.00E-41	100	QKK83122.1
21	15262-15687	426	141	Hypothetical protein [*Vibrio* phage vB_VpaP_MGD1]	100	4.00E-95	100	QKK83123.1
22	15690-16640	951	316	Exonuclease [*Vibrio* phage vB_VpP_NS8]	100	0	100	QYW05858.1
23	16627-16833	207	68	Hypothetical protein [*Vibrio* phage vB_VpP_FE11]	100	9.00E-42	100	QIW87167.1
24	16820-17260	441	146	Endonuclease [*Vibrio* phage BUCT233]	100	6.00E-102	100	QWE49801.1
25	17260-17379	120	39	Hypothetical protein [*Vibrio* phage vB_VpaP_KF2]	100	8.00E-20	100	YP_009808096.1

Table S1 Annotation of *Vibrio* phage VPK8 genome. The open reading frames (ORFs) and representative sequences were analyzed through homology searches against protein entries in the GenBank database using BLASTP (Continued).

ORF	Genomic coordinates	Length (bp)	Amino acid	Representative seq [Organism]	Coverage (%)	*E*-value	Identity (%)	Accession no.
26	17376-17960	585	194	Putative deoxynucleoside monophosphate kinase [*Vibrio* phage vB_VpP_DE18]	100	1.00E-138	98.97	QWY13564.1
27	18146-20596	2451	816	DNA-directed RNA polymerase [*Vibrio* phage BUCT233]	100	0	99.63	QWE49798.1
28	20627-21046	420	139	GNAT family N-acetyltransferase [*Vibrio* phage vB_VpaS_OWB]	100	7.00E-99	100	YP_009948734.1
29	21033-21278	246	81	Hypothetical protein [*Vibrio* phage VP93]	100	3.00E-48	100	YP_002875650.1
30	21287-22819	1533	510	Putative head-tail connector protein [*Vibrio* phage vB_VpaP_MGD1]	100	0	100	QKK83133.1
31	22819-23634	816	271	Putative scaffolding protein [*Vibrio* phage vB_VpP_HA5]	100	0	100	UXF57368.1
32	23699-24697	999	332	Major capsid protein [*Vibrio* phage vB_VpP_DE10]	100	0	100	QXV72174.1
33	24709-24837	129	42	Hypothetical protein [*Vibrio* phage F23s2]	100	1.00E-20	100	UPT53620.1

Table S1 Annotation of *Vibrio* phage VPK8 genome. The open reading frames (ORFs) and representative sequences were analyzed through homology searches against protein entries in the GenBank database using BLASTP (Continued).

ORF	Genomic coordinates	Length (bp)	Amino acid	Representative seq [Organism]	Coverage (%)	*E*-value	Identity (%)	Accession no.
34	24903-25463	561	186	Putative tail tubular protein A [*Vibrio* phage vB_VpaP_MGD1]	100	1.00E-134	100	QKK83137.1
35	25473-27815	2343	780	Non-contractile tail tubular protein [*Vibrio* phage vB_VpP_DE10]	100	0	99.36	QXV72177.1
36	27825-28571	747	248	Internal virion protein [*Vibrio* phage vB_VpP_HA1]	100	2.00E-173	99.19	UXF57316.1
37	28581-31259	2679	892	Internal virion protein [*Vibrio* phage vB_VpP_HA1]	100	0	99.66	UXF57315.1
38	31309-35163	3855	1284	Peptidoglycan lytic exotransglycosylase [*Vibrio* phage vB_VpP_HA5]	100	0	99.69	UXF57361.1
39	35185-35796	612	203	Tail fibers protein [*Vibrio* phage vB_VpP_DE10]	100	1.00E-145	99.01	QXV72181.1
40	35805-38537	2733	910	Putative glycosyl hydrolase [*Vibrio* phage vB_VpP_HA5]	100	0	99.34	UXF57406.1
41	38547-38846	300	99	Terminase small subunit [*Vibrio* phage VP93]	100	5.00E-60	100	YP_002875661.1

Table S1 Annotation of *Vibrio* phage VPK8 genome. The open reading frames (ORFs) and representative sequences were analyzed through homology searches against protein entries in the GenBank database using BLASTP (Continued).

ORF	Genomic coordinates	Length (bp)	Amino acid	Representative seq [Organism]	Coverage (%)	*E*-value	Identity (%)	Accession no.
42	38857-40776	1920	639	Terminase large subunit [*Vibrio* phage BUCT233]	100	0	100	QWE49783.1
43	40773-40865	93	30	Hypothetical protein [*Vibrio* phage vB_VpP_FE11]	100	1.00E-25	100	QIW87186.1
44	40881-41180	300	99	Hypothetical protein [*Vibrio* phage VP93]	100	5.00E-63	100	YP_002875663.1
45	41189-41746	558	185	Hypothetical protein [*Vibrio* phage vB_VpaP_MGD1]	100	3.00E-129	100	QKK83147.1
46	41768-42181	414	137	Endolysin [*Vibrio* phage vB_VpaP_KF1]	100	2.00E-95	98.54	YP_009808038.1
47	42174-42530	357	118	Hypothetical protein [*Vibrio* phage vB_VpaP_KF1]	100	3.00E-80	99.15	YP_009808039.1
48	42584-42865	282	94	Hypothetical protein [*Vibrio* phage vB_VpP_FE11]	79	6.00E-34	85.33	QIW87192.1
